# Talipes equinovarus or Clubfoot: A review of study approaches, management and trends in Pakistan

**DOI:** 10.12669/pjms.36.6.2514

**Published:** 2020

**Authors:** Khadija Murtaza, Zahra Saleem, Sajid Malik

**Affiliations:** 1 Khadija Murtaza, Human Genetics Program, Department of Zoology, Faculty of Biological Sciences, Quaid-i-Azam University, Islamabad 45320, Pakistan; 2 Zahra Saleem, Human Genetics Program, Department of Zoology, Faculty of Biological Sciences, Quaid-i-Azam University, Islamabad 45320, Pakistan; 3 Sajid Malik, Human Genetics Program, Department of Zoology, Faculty of Biological Sciences, Quaid-i-Azam University, Islamabad 45320, Pakistan

**Keywords:** Idiopathic clubfoot, Congenital talipes equinovarus, Foot anomaly, Ponseti method

## Abstract

This paper aims to assess the status of scientific literature on talipes equinovarus (TEV) published from Pakistan, to get an insight into the trend in knowledge over the years, and to highlight study gaps in this area. A detailed review of published literature was conducted from November 2019 to January 2020. ‘Talipes/congenital talipes’, ‘clubfoot/congenital clubfoot’, ‘talipes equinovarus /congenital talipes equinovarus’ AND ‘Pakistan’ were used as key terms. Different search engines, PubMed, PakMediNet, ScienceDirect, Embase and Google Scholar were utilized to retrieve articles. A total of 63 articles were retrieved. The hotspot of TEV research in Pakistan has been its treatment and management. Over the years, treatment trend has shifted from operative to conservative; Ponseti method is predominantly employed. Hospital-based studies focusing on pediatric patients are common while population-based studies are devoid. In majority of cohorts, there is preponderance of male patients, idiopathic and unilateral cases. There is, however, scarcity of basic research on the prevalence, etiology, risk factors, clinical heterogeneity, associated anomalies, genetics, and molecular diagnostics of TEV. In conclusion, prudent scientific evidence is required for any policy-making and relevant public health action. Hence, large scale population-based studies are required for a broader overview and understanding the clinical spectrum of TEV.

## INTRODUCTION

Talipes equinovarus or clubfoot (OMIM-119800) is a gross deformity of the foot presented at birth.^1^ The word *talipes* is derived from *talus* (ankle) and *pes* (foot). Talipes denotes the club-like appearance of the foot and exists in various subtypes; talipes equinovarus (TEV) being the most prevalent one. The incidence of TEV is about 1/1000 live births per year. It is the seventh most prevalent congenital birth anomaly and the most common of the musculoskeletal system. Globally the burden of this birth defect affects more than 150,000 infants every year.^2^,^3^ Among all the cases born worldwide, 80% live in low- and middle-income countries.^4^ It is depicted in 5,000 years old Egyptian hieroglyphs and firstly reported by Hippocrates 400 years BC.^5^

TEV can occur as an isolated entity, usually termed as idiopathic, or as a syndromic condition. In its syndromic presentation, it arises in many neurological, neuromuscular and paralytic disorders.^5^ Both idiopathic and syndromic conditions can be milder or severe. It generally has a sporadic occurrence but familial cases showing segregation in several generations are also reported. Its etiology is considered to be a combination of genetic and environmental factors.^6^ TEV has a highly negative impact on the life of the subject. If left untreated it may result in dependency on others for performing the daily activities, resulting in heavy economic burden on the family and the country.^7^

For prenatal diagnosis, ultrasonography is considered the most reliable and majority of the cases can be diagnosed after 17 weeks of gestation. Treatment of TEV comprises both surgical and non-surgical methods and is effective in the early years of life. The Ponseti method remains the most popular non-surgical technique.^8^

The present study was aimed to assess the status of scientific literature on TEV published from Pakistan, to get an insight into the trends in knowledge over the years, and to highlight the study gaps in this area, hence to provide directions for further research.

## METHODS

A review of the literature was conducted from November-2019 to January-2020 and all the papers fulfilling the inclusion criteria and published by the Pakistani researchers were considered. The search strategy adopted was an article title/keyword/abstract-based search using the following key terms: ‘talipes/congenital talipes’, ‘clubfoot/congenital clubfoot’, ‘talipes equinovarus/congenital talipes equinovarus’ in Pakistan. TEV reported under the study title of birth defects, musculoskeletal disorder, and congenital foot deformities, were included. PubMed, PakMediNet, Medline, Embase, Science Direct, and Google Scholar were the search engines employed for literature search. The pertinent information including authors, institute, study setting, duration, sample size, target population, age group, goals, and management approach, was extracted. Data were maintained in Excel sheet.

## RESULTS

### Journals, time era and study setting

A total of 63 articles were retrieved; of these 56 (89%) studies were published in local journals while 7 (11%) were published in international journals. The highest number of studies (n=10) were published in J Pak Orthop Assoc.

**Fig.1:**
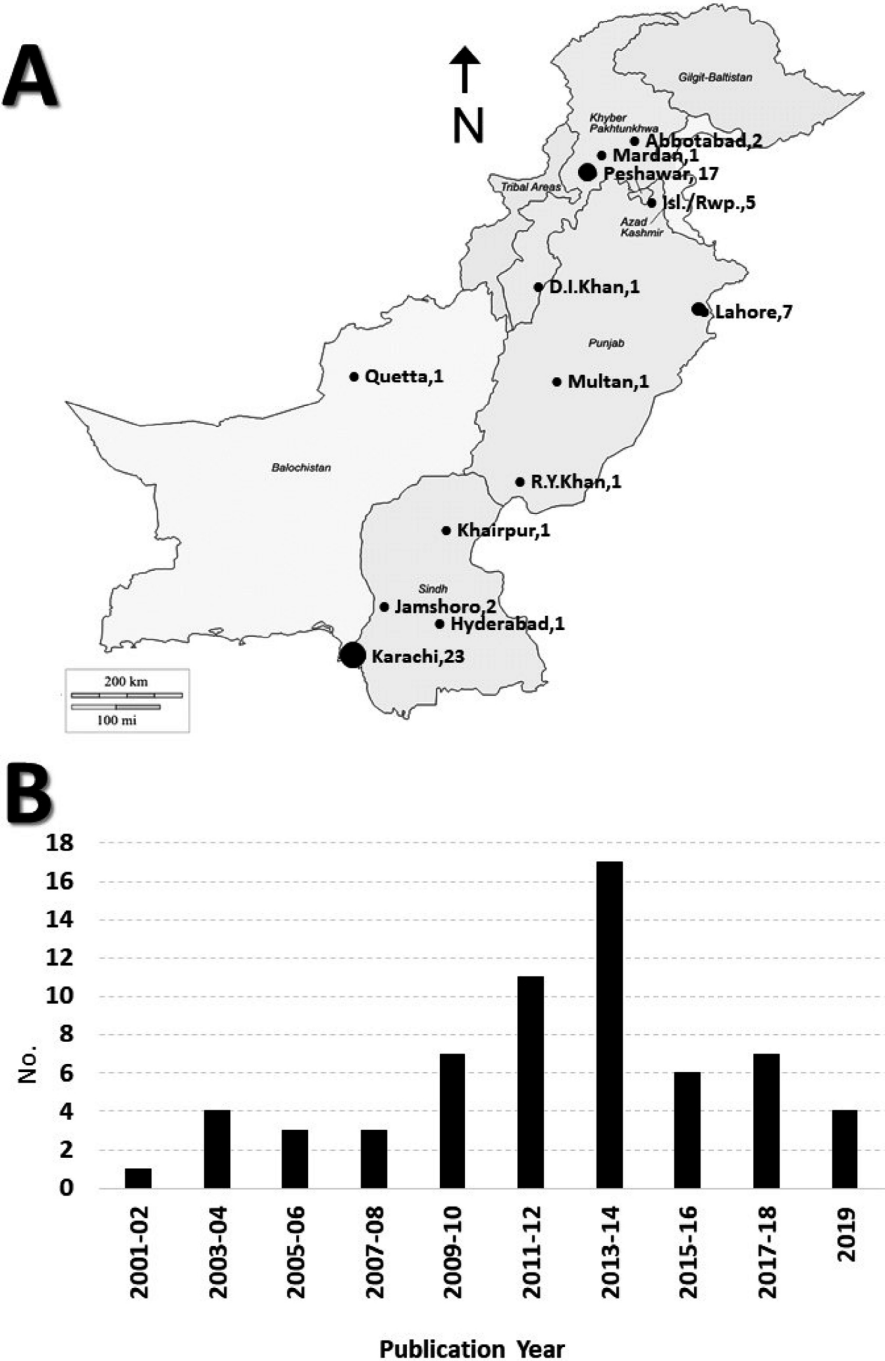
A. Mapping of number of studies on TEV published from various cities. B. Number of studies published on TEV depicted on bi-annual bar-chart.

Extensive studies were conducted during the period 2011-2014 (n=28), followed by 2015-2019 (n=17). The highest number of studies were conducted in Sindh (n=27), followed by Khyber Pakhtunkhwa (n=21) and Punjab (n=14) (Fig.1).

### Incidence, prevalence and epidemiology

The studies reporting true birth-prevalence of TEV in Pakistan are scarce. Its estimated incidence was 6,000–7,000 cases/year; i.e., 1.4:1,000 livebirths and 1.5/1,000 livebirths.^9^,^10^

### Study cohorts, age and gender distribution

The majority of the reported studies are prospective cross-sectional or descriptive case-series (Table-I). The study cohorts were mostly pediatric population. The male subjects were highly represented in most of the studies (70%). Only two studies reported a high representation of female patients.^11^,^12^

**Table-I:** Summary of representative studies carried out in Pakistan on TEV.

Reference	Institute	Design	Duration	Sample	Study domain	Theme/management plans
Din, 2004(39)	Hayatabad Med Complex, Peshawar	Prospective	1998-2000	96	Non-operative	Kite-Lovell technique
Khan and Chinoy, 2006(15)	Karachi	Prospective	2000-2004	15	Operative	Double zigzag incision as single-stage procedure; neglected cases
Humail et al. 2009(16)	Dow Uni of Health Science, Karachi	Prospective-descriptive	1998-2004	360	Operative & Non-oper.	Turcos procedure, serial casting, neglected cases
Ishaque, 2009(25)	Baqai Med Uni, Karachi	Review	--	--		Conservative management
Sami et al. 2010(38)	Mayo Hospital, Lahore	Cross-sectional	18 months	50	Non-operative	Case history, clinical parameters
Ahmed et al. 2011(12)	Liaquat Uni of Med Sci, Jamshoro	Prospective-descriptive	2005, 2009	20	Operative	Split tibialis anterior and posterior tendon transfer
Jalil et al. 2011(20)	Abbasi Shaheed Hospital, Karachi	Retrospective, descriptive	2006-2008	13	Operative	Revision surgery, PMR, Turco’s, neglected/relapsed cases
Makhdoom et al. 2011(21)	Liaqat Uni of Med Sci, Jamshoro	Observational-descriptive	2007-2010	49	Non-operative	Ponseti method
Inam et al. 2012(11)	Hayatabad Med Complex, Peshawar	Comparative	2008-2010	60	Operative & Non-oper.	Ponseti vs.Turco’s posteromedial
Khan et al. 2012(37)	Khyber Teaching Hospital, Peshawar	Prospective	2008-2010	45	Operative	One stage posteromedial release
Akhter et al. 2013(14)	PIMS, Islamabad	Retrospective	2008-2011	23	Operative	Percutaneous tendo Achilles lengthening
Irfan and Mehboob, 2013(27)	MultiCenters, Lahore	Observational	Over 3 years	1000 expecting mothers	Non-operative	Prenatal ultrasonographic detection
Khan et al. 2013(36)	Khyber Teaching Hospital, Peshawar	Cross-sectional	2009-2010	70	Non-operative	Ponseti method
Zia et al. 2013(35)	Benazir Bhutto Hospital, Rawalpindi	Prospective case series	2010-2011	55	Non-operative	Ponseti method
Hussain et al. 2014(10)	Indus Hospital, Karachi	Descriptive	2012	Parents	Non-operative	Cost-effectiveness of Ponseti
Khan et al. 2014(34)	Khyber Teaching Hospital, Peshawar	--	2009-2010	70	Non-operative	Achilles tendon tenotomy in Ponseti
Memon et al. 2014(33)	Jinnah Postgrad. Med Centre, Karachi	Cross-sectional	2012-2013	125	Non-operative	Ponseti method
Ullah et al. 2014(18)	Hayatabad Med Complex, Peshawar	Prospective experimental	2013-2014	28	Non-operative	Accelerated Ponseti, neglected cases
Aftab and Khan, 2015(28)	PIPOS, Peshawar	Retrospective	2014	30	Non-operative	Ponseti method
Bhatti et al. 2015(9)	Jinnah Postgrad. Med Centre, Karachi	Descriptive case series	2013	200	Natural history	Risk factors
Iqbal et al. 2015(32)	Sheikh Zayed Hospital, Rahim Yar Khan	Descriptive case series	2012	146	Non-operative	Ponseti method
Ihsanullah et al. 2016(31)	Hayatabad Med Complex, Peshawar	Cross-sectional	2014-2015	144	Natural history	Dysplasia of hip in children with TEV
Khan et al. 2017(30)	Indus Hospital, Karachi	Descriptive case series	2011-2016	706	Non-operative	Pirani scoring
Rashid et al 2017(22)	Children Hospital, Lahore	Retrospective	--	67	Non-operative	Foot abduction orthosis, relapsed idiopathic
Shah et al. 2017(26)	Ayub Teaching Hospital, Abbottabad	Descriptive case series	2015-2016	177	Non-operative	Ponseti method
Akram et al. 2018(29)	PIPOS, Peshawar	Descriptive cross-sectional	2014	107	Natural history	Risk factors
Ullah and Shah, 2018(19)	Lady Reading Hospital, Peshawar	Case study	--	1	Non-operative	Ponseti method, neglected cases
Ahmed et al. 2019(24)	Ghurki Trust Teaching Hospital, Lahore	Randomized controlled trial	2017-2019	80	Non-operative	Classical vs. Accelerated Ponseti
Jamil et al. 2019(17)	Dr Ruth Pfau Civil Hospital, Karachi	Retrospective cross-sectional	2013-2016	28	Non-operative	Ponseti method, neglected cases
Kashif et. al 2019(23)	Mercy Teaching Hospital, Peshawar	Descriptive	2015, 2018	46	Natural history	Causes of neglected/relapsed cases

### Clinical and phenotypic attributes

*Talipes equinovarus* (TEV) is the only clinical type reported in Pakistani literature. The International Classification of Disease (ICD-10) database presents at least 9 talipes variants namely *talipes equinovarus* (Q66.0), *talipes calcaneovarus* (Q66.1), *metatarsus varus* (Q66.2), *talipes varus* (Q66.3), *talipes calcaneovalgus* (Q66.4), *talipes planus* (Q66.5), *talipes valgus* (Q66.6), *talipes cavus* (Q66.7), and *talipes equinus* (Q66.8).^13^ Even though TEV has been presented as the most prevalent variant yet the relative prevalence of other variants remains unknown.

The unilateral cases were presented in higher preponderance as compared to bilateral. Most of the studies dealt with idiopathic TEV as compared to non-idiopathic. Three studies reported treatment cohorts of paralytic deformities causing foot anomaly; i.e., cerebral palsy,^12^ cerebral palsy and poliomyelitis,^14^ arthrogryphosis multiplex congenita.^15^ Only five studies dealt with neglected cases.^15^-^19^ Resistant/relapsed cases were studied in four articles (Table-I).^20^-^23^

### Genetics

Indeed, no study has been published from Pakistan which could highlight the genetic underpinning of TEV. Hence, information regarding the inheritance pattern(s) of TEV, its genetic mapping, gene identification, association studies showing risk SNPs, twin studies and effect of consanguinity and familial attributes, remain to be elucidated in multi-ethnic/multi-lingual Pakistani cohorts.

### Treatment and management of TEV

Most of the reported studies were hospital-based focusing on the treatment-seeking group while community-based studies were deficient. The studies on non-operative management were more common as compared to operative management (31 vs. 19, respectively). It was quite evident that the trend of studies over the years has shifted from operative to conservative management. During 2000-2004 and 2005-2009 the ratio of papers reporting operative-to-conservative was (2:2) and (6:1), respectively, while in 2010-2014 and 2015-2019, the ratio is (10:19) and (1:13), respectively. The latest studies majorly covered the conservative treatment domain.

Various conservative treatment methods were in practice in Pakistan. Before 2006, Kite method and surgical treatment were in practice. Later, Ponseti and accelerated Ponseti methods became popular.^18^,^24^ However, no study was reported regarding the French method. Ishaque (2009) has reviewed the conservative management of TEV.^25^

Studies also reported various surgical measures, i.e., Turco procedure, Window procedure, Posteromedial release, Subtalar release, Modified Turco’s Postero-Medial release, etc. Pirani scoring was commonly used to assess the severity level. Irfan and Mehboob (2013) carried out ultrasonography for prenatal detection of TEV in 1,000 expecting women in Lahore.^27^

## DISCUSSION

To the best of our knowledge, it is the first review that assembled the available Pakistani literature on TEV regarding its natural history, study designs and management. The chronological arrangement of published reports has revealed that this malformation has gained some attention among the researchers over the years. However, the researchers have predominantly explored a relatively narrow domain and many of the fundamental questions on TEV remain to be elucidated (see below). There has been no comprehensive study reporting the true prevalence and incidence of TEV,^28^ thus the burden of this disorder remains unknown. Traditionally, the hospital-based studies mainly focus on the treatment-seeking group while the pattern of malformation in the general population remains obscure. Further, most of the literature has been published from Khyber Pakhtunkhwa and Sindh provinces and the representative data from the tertiary care institutes of other cosmopolitan cities across Pakistan are deficient.

Here, the researchers have primarily focused on the treatment/management of TEV. Both conservative and operative management have been practiced, however, the trend has shifted from operative to conservative management (Table-I). Follow up studies have been conducted, but short-term follow up should be complemented with long-term follow up in order to assess treatment outcome.^26^ Mismanagement, noncompliance, natural history and severity of disease are responsible for the recurrence.^23^,^40^

The younger/pediatric population remains the focus of most of the studies. There are several potential hurdles in Pakistan including poverty, lack of awareness, lack of specialized clinics/doctors, that delay the treatment of TEV at younger ages. Hence, the neglected or late cases are overrepresented, culminating an increased prevalence of this malformation.^16^ Interestingly, unilateral cases were highly presented in majority of the reported cohorts which is contrasting to the other studies where bilateral TEV cases comprised 50% of samples.^8^,^41^ Hence, investigations are required in order to understand the underlying cause(s) of this discrepancy. Bilateral clubfoot may result from an increased load of genetic factors.^5^,^6^

The etiology of TEV includes both genetic as well as environmental factors. Genetics has a clear causative role in a substantial number of TEV cases.^2^ So far, no study was reported from Pakistan concerning gene identification, gene mapping, genetic mutation and mode of inheritance. Family history, consanguinity, familial attributes, etc. were evaluated in only a few studies.^9^,^10^,^30^,^33^ In familial cases, the degree of relatedness plays a key role as first degree relatives are more prone to disorder as compared to distant relatives;^42^ these factors are largely unaddressed in Pakistan. Besides this, studies reporting the role of maternal, and environmental factors and seasonal variation are scarce.^9^,^33^ Further, TEV has 33% concordance among monozygotic twins while 3% in dizygotic twins.^42^ This area also remains to be explored in Pakistani cohorts.

Clubfoot has a negative impact on the life of patient; if it is left untreated it may cause dependency on others for performing the daily activities, difficulties in ambulation and lifelong disability. It causes heavy economic burden not only on the family but on the country as well. An estimated 80% of such cases can be seen in developing countries.^43^

### Limitations of the study

All of the clinical parameters of TEV reported in the literature were not covered due to limitation of space. Studies on clubfoot related to prenatal ultrasonographic detection; awareness, perception and attitude towards clubfoot; perspectives from the caregivers’ standpoint; laboratory/motor electrophysiological studies, and surgical approaches, were not included.

## CONCLUSION

The hotspot of TEV research in Pakistan is its treatment and management, predominantly the Ponseti method. This study reveals that there is a scarcity of research on various important aspects of TEV in Pakistan, and its epidemiology, prevalence, etiology, risk factors, associated anomalies, maternal and obstetric factors, birth parameters, molecular diagnostics, etc., need to be elucidated. Moreover, large scale population-based studies are required for a broader overview of the malformation. This review highlights marked dearth of scientific evidence on TEV required for awareness, policy-making and relevant public health action.

### Authors’ Contribution:

**SM** conceived, designed and planned study and also responsible and accountable for the accuracy or integrity of the work.

**KM & ZS** did data collection and manuscript writing.

**KM, ZS & SM** edited, reviewed and approval manuscript.
